# Expression and Critical Role of Interleukin Enhancer Binding Factor 2 in Hepatocellular Carcinoma

**DOI:** 10.3390/ijms17081373

**Published:** 2016-08-22

**Authors:** Shaobing Cheng, Xu Jiang, Chaofeng Ding, Chengli Du, Kwabena Gyabaah Owusu-Ansah, Xiaoyu Weng, Wendi Hu, Chuanhui Peng, Zhen Lv, Rongliang Tong, Heng Xiao, Haiyang Xie, Lin Zhou, Jian Wu, Shusen Zheng

**Affiliations:** 1 Department of Hepatobiliary Surgery, The First Affiliated Hospital, Zhejiang University School of Medicine, Hangzhou 310003, China; showcheng@126.com (S.C.); drjiangxu@126.com (X.J.); dcf25@126.com (C.Di.); 496722098@163.com (C.Du); Kobby01@live.com (K.G.O.-A.); wengxiaoyu2009@sina.com (X.W.); huwendi1988@gmail.com (W.H.); clarkpch@163.com (C.P.); lvzhen@zju.edu.cn (Z.L.); trl55555@126.com (R.T.); xiaohengdoctor@126.com (H.Xia.); xiehy@zju.edu.cn (H.Xie); linzhou19@163.com (L.Z.); 2 Collaborative Innovation Center for Diagnosis and Treatment of Infectious Diseases, The First Affiliated Hospital, Zhejiang University School of Medicine, Hangzhou 310003, China; 3 Key Laboratory of Combined Multi-organ Transplantation, Ministry of Public Health, Key Laboratory of Organ Transplantation, Hangzhou 310003, China

**Keywords:** interleukin enhancer binding factor 2, hepatocellular carcinoma, cell growth, apoptosis

## Abstract

Interleukin enhancer binding factor 2 (ILF2), a transcription factor, regulates cell growth by inhibiting the stabilization of mRNA. Currently, its role has gained recognition as a factor in the tumorigenic process. However, until now, little has been known about the detailed role ILF2 plays in hepatocellular carcinoma (HCC). In this study, we investigated the expression levels of ILF2 in HCC tissue with Western blot and immunohistochemical assays. To examine the effect of ILF2 on liver cancer cell growth and apoptosis, small interfering RNAs (siRNAs) targeting ILF2 were recombined to create lentiviral overexpression vectors. Our results showed higher expression levels of ILF2 mRNA and ILF2 protein in HCC tissue compared with matched peritumoral tissue. Expression of ILF2 may regulate cell growth and apoptosis in liver cancer cells via regulation of B-cell lymphoma 2 (Bcl-2), Bcl-2 related ovarian killer (Bok), Bcl-2-associated X protein (BAX), and cellular inhibitor of apoptosis 1 (cIAP1). Moreover, we inoculated nude mice with liver cancer cells to investigate the effect of ILF2 on tumorigenesis in vivo. As expected, a rapid growth was observed in cancer cells inoculated with a lentiviral vector coding Flag-ILF2 (Lenti-ILF2) compared with the control cells. Hence, these results promote a better understanding of ILF2’s potential role as a therapeutic target in HCC.

## 1. Introduction

Hepatocellular carcinoma (HCC) is one of the most common solid tumors, with an estimated 782,500 new cases and 745,500 deaths reported in 2012 worldwide [1]. Although much progress has been made in treatment options for patients with HCC, surgery remains the main treatment option for such patients, with a five-year survival rate of about 30%–40% [2]. Other treatment options, such as radiation therapy, radiofrequency ablation, transarterial chemoembolization, and liver transplantation, have been performed in some HCC patients when tumor resection was difficult [3]. Patients with HCC show poor prognosis, in part due to the lack of effective therapeutic options. Hence, determination of the genes and molecular mechanisms involved in HCC is considered extremely urgent for improving the diagnosis of multiple stages of HCC and facilitating drug development with specific liver cancer targets.

Interleukin enhancer binding factor 2 (ILF2), also known as nuclear factor 45 (NF45), is encoded by a gene located on human chromosome 1 (1q11-qter and 1p11-p12). It is confirmed by polymerase chain reaction (PCR) amplification of ILF2-specific DNA sequences [4], and is consistent with a prior localization of the NF45 gene to chromosome 1q21.3 by fluorescence in situ hybridization (FISH) [5]. ILF2/NF45 associates with ILF3/NF90 in the nucleus and regulates *IL-2* gene transcription at the antigen receptor response element (ARRE)/nuclear factor of activated T-cells (NFAT) DNA target sequence [6]. Also, cells deficient in ILF2 exhibit reduced internal ribosome entry site (IRES)-mediated translation of X-linked inhibitor of apoptosis protein (XIAP) and cellular inhibitor of apoptosis protein 1 (cIAP1) [7,8]. Furthermore, knockdown of NF90 destabilized NF45, and vice versa. Similarly, NF110 and NF45 are coregulated [5]. ILF2/NF45 was shown to be regulated by meiosis and is associated with transcriptionally active chromatin [9]. In addition to its potential role in regulating transcription, NF45 may be associated with RNAs in ribonucleoprotein complexes that regulate delayed translation of mRNAs [10,11]. NF45 may directly bind to pri-miRNAs to reduce miRNA production by hindering the accessibility of the microprocessor complex [12]. Cotransfection of ILF2 and ILF3 has been demonstrated to augment transcriptional activation. NF45 has also been identified as a component of the spliceosome [13,14]. The recovery of ILF2 in the ribosomal salt wash and the physical association with protein kinase R (PKR) suggest that ILF2 is involved in regulating translation [15,16,17]. A growing pool of evidence has indicated that overexpression of ILF2 is frequently observed in glioma, non-small cell lung cancer, esophageal squamous cell carcinoma, and childhood endodermal sinus tumors and is related to poor clinical outcomes [18,19,20,21]. Results on the induction of p53 and p21 by knockdown of ILF2 in human cervical carcinoma cells have recently been reported [22]. Upregulation of ILF2 in HCC has been validated by Western blot and immunohistochemistry [23]. Although the regulation and function of ILF2 have been extensively investigated, the biological functions as well as the molecular mechanisms of ILF2 in tumorigenesis and tumor progression have not been fully demonstrated.

In this study, we discovered the aberrant expression of ILF2 in HCC, and its regulation of liver cancer cell proliferation, cell growth, and apoptosis. Furthermore, we discovered that ILF2 promotes cell proliferation and enhances the tumorigenic capacity in vivo. Our data revealed that ILF2 might play a crucial role in liver carcinogenesis and serve as a potential target for HCC therapy.

## 2. Results

### 2.1. Interleukin Enhancer Binding Factor 2 Is Upregulated in Human Hepatocellular Carcinoma

**Figure 1:**
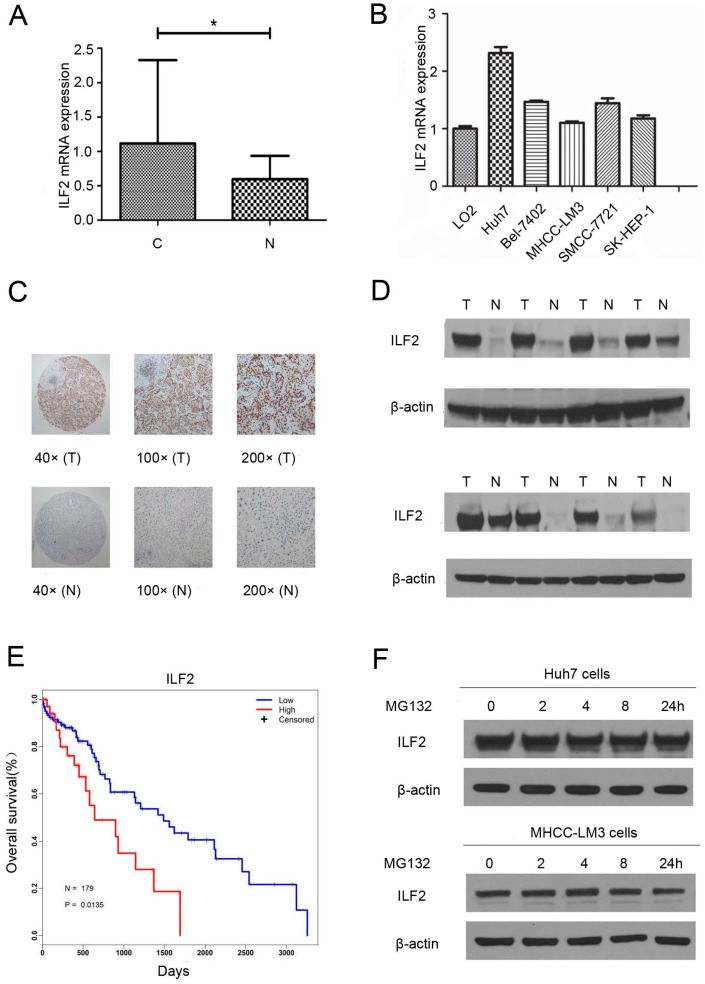
Expression patterns of interleukin enhancer binding factor 2 (ILF2) in hepatocellular carcinoma (HCC) tissues and cell lines. (**A**,**B**) mRNA levels of ILF2 were analyzed using quantitative real-time PCR (qRT-PCR) in HCC tissues and HCC cell lines; bars, standard deviation (SD) * *p* < 0.05; (**C**) Immunohistochemical image of ILF2 expression in liver tumor tissues (T) and normal tissues (N). Representative image of ILF2 at 40×, 100×, and 200× magnification; (**D**) ILF2 protein expression in HCC (T) and normal (N) tissues; (**E**) Kaplan–Meier survival curves of overall survival of HCC patients with high and low ILF2 expression; *p* = 0.0135; Regression coefficient (Coef) = 0.7187; Hazard ratio exp(coef) = 2.0518; 95% lower confidence limits (lower 0.95) = 1.1453; 95% upper confidence limits (upper 0.95) = 3.6757; (**F**) Huh7 and MHCC-LM3 cells following treatment with 10 μM MG132 at indicated time points. ILF2 and β-actin levels were measured by Western blot.

First, ILF2 expression was analyzed in 27 paired HCC and corresponding neighboring normal tissues using quantitative real-time PCR (qRT-PCR). ILF2 was found to be upregulated in HCC tissues (*p* < 0.05, Figure 1A). As shown in Figure 1B, higher levels of ILF2 were observed in liver cancer cell lines than in a normal liver cell line (LO2). To confirm the qRT-PCR results, we first measured ILF2 expression in 72 paired HCC tissues by immunohistochemical assay. The results showed higher expression levels in HCC compared with adjacent non-cancerous tissues (Figure 1C). Moreover, the results also revealed that expression of ILF2 was correlated with tumor size (*p* = 0.043) (Table 1). In agreement with the above results in HCC, ILF2 protein expression was upregulated in HCC as assessed by Western blot (Figure 1D). In order to determine the impact of high ILF2 expression levels in the prognosis of HCC patients, the Kaplan–Meier method was used to compare the prognosis among HCC patients with high and low ILF2 expression levels in The Cancer Genome Atlas (TCGA) dataset [24]. We analyzed the differences in the overall survival after surgery between high and low ILF2 expression groups. Interestingly, HCC patients with high ILF2 expression levels experienced shorter survival time compared with the low expression group by TCGA data analysis (*p* = 0.0135) (Figure 1E). The first quartile was used to define the high ILF2 expression group and low ILF2 expression group, with 4287.62 as the cut-off value. To determine whether ILF2 upregulation occurs via transcription and/or degradation, we used the proteasome inhibitor MG132. The cells were incubated with MG132 several times, after which cell lysates were subjected to immunoblot analysis. No significant changes in the level of ILF2 were observed in HCC cell lines (Huh7 and MHCC-LM3) treated with MG132 (Figure 1F). Therefore, our results indicate that ILF2 expression is upregulated in both HCC tissues and liver cancer cell lines, and upregulation of ILF2 occurs via transcription and not via protein turnover.

### 2.2. Effects of Interleukin Enhancer Binding Factor 2 on the Proliferation of Liver Cancer Cells

**Figure 2:**
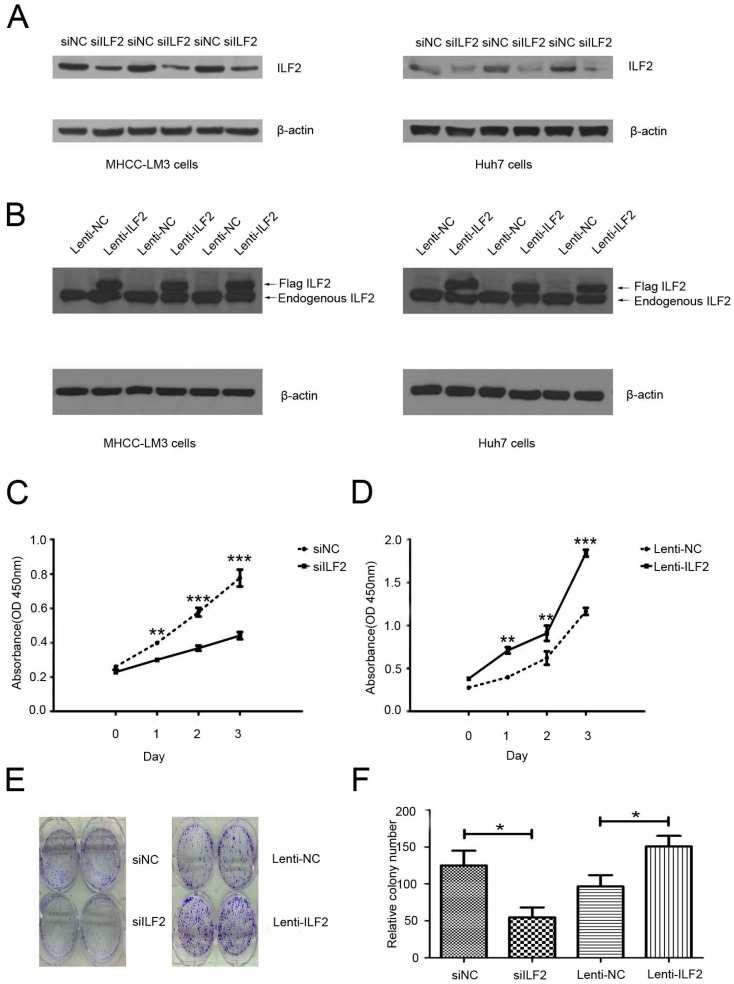
Effects of ILF2 silencing and overexpression on liver cancer cell proliferation. (**A**) Downregulation of ILF2 expression by small interfering RNA (siRNA) in Huh7 and MHCC-LM3 cells; (**B**) ILF2 expression in Huh7 and MHCC-LM3 cells infected with recombinant ILF2 lentivirus; (**C**,**D**) proliferation ability of cells in vitro after transfection with siRNA. Infection with recombinant lentivirus was evaluated by Cell Counting Kit-8 (CCK-8) assay. Data shown as mean (*n* = 3) ± SD ** *p* < 0.01, *** *p* < 0.001; (**E**) Representative images of the colony formation assay in Huh7 and MHCC-LM3 cells; (**F**) Quantification of colony number. Data shown as mean (*n* = 3) ± SD * *p* < 0.05. siILF2: ILF2 siRNA; siNC: siRNA negative control.

To explore its potential role in HCC proliferation, we utilized small interfering RNA (siRNA) transfection and lentivirus overexpression methods to remove the endogenous ILF2 and enhance the expression of exogenous ILF2 in liver cancer cells, respectively. HuH7 and MHCC-LM3 cells were transduced with a lentiviral vector coding Flag-ILF2 (Lenti-ILF2) and a lentiviral vector negative control (Lenti-NC). Based on the expression patterns of ILF2 in the liver cancer cell lines, MHCC-LM3 and Huh7 were selected to perform the experiments. The levels of endogenous ILF2 knockdown and Flag-ILF2 in Huh7 and MHCC-LM3 cells were validated (Figure 2A,B). For objective quantification of bands, we used densitometry with the ImageJ 1.44 software [25]. As shown in Figure S1, Western blot densitometric analysis allowed quantification of ILF2 protein bands in comparison to β-actin. Notably, since lentiviral vectors carrying Flag-ILF2 or a negative control vector and corresponding viruses at 1 × 10^8^ plaque forming units (pfu)/mL were constructed in Lenti-ILF2 or Lenti-NC cells, whole-cell extracts were used for anti-Flag immunoprecipitation, consistent with previous results. Flag-ILF2 was detected by Western blot in the Lenti-ILF2 group but not in the Lenti-NC group (Figure S2). Next, we demonstrated the effects of ILF2 silencing and overexpression on the proliferation of liver cancer cells by Cell Counting Kit-8 (CCK-8) assay and colony formation assay. While negative control transfected cells grow rapidly, ILF2-depleted cells display a slow rate of proliferation, as shown in the CCK-8 assay (Figure 2C). Conversely, the proliferation rate of cells transfected with Lenti-ILF2 was significantly faster than that in cells treated with the Lenti-NC (Figure 2D). Similarly, the results were also validated from the colony formation assay in Huh7 and MHCC-LM3 cells (Figure 2E,F). Data presented here indicate that ILF2 plays a significant role in liver cancer cell growth, cell proliferation, and survival.

### 2.3. Effects of Interleukin Enhancer Binding Factor 2 on Apoptosis of Liver Cancer Cells

**Figure 3:**
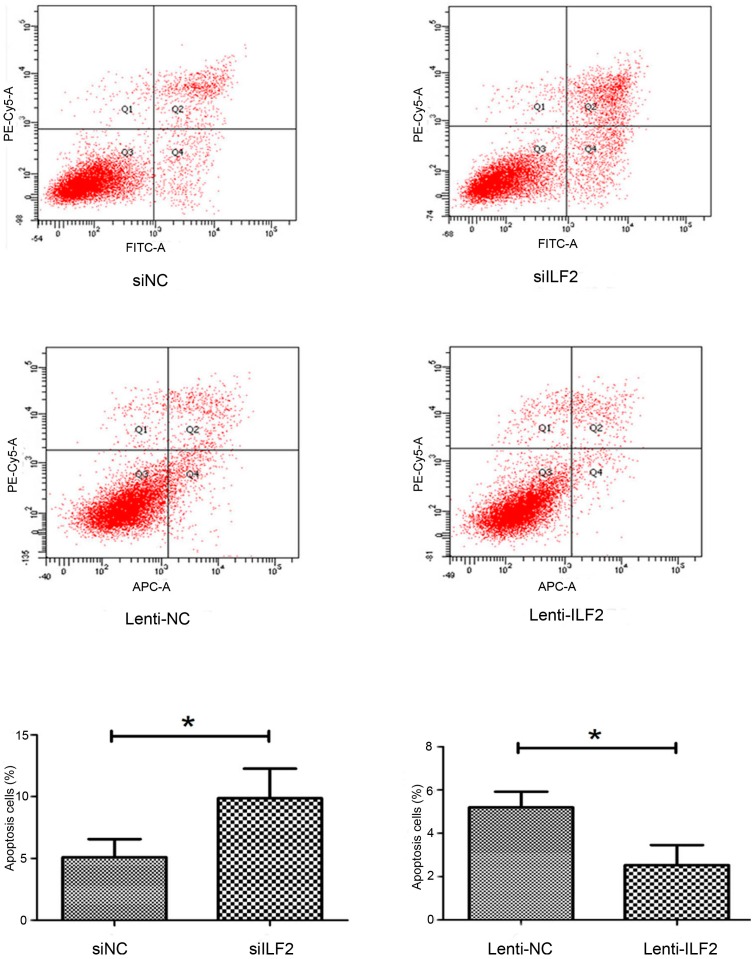
Effects of ILF2 knockdown and overexpression on apoptosis. Cells were transfected with siRNAs (siNC and siILF2) and infected with recombinant lentivirus (Lenti-NC and Lenti-ILF2). Annexin V-fluorescein isothiocyanate (FITC)/propidium iodide (PI) assay for determination of apoptosis was then performed. Cells were analyzed by flow cytometry. Representative images of each group are shown. Data shown as mean (*n* = 3) ± SD, * *p* < 0.05.

Next, to determine whether the increase in cell proliferation was due to inhibition of apoptosis and how ILF2 expression correlates with apoptosis in liver cancer cells, we performed an apoptosis assay by flow cytometry. As expected, ILF2 overexpression significantly decreased apoptosis in cancer cells infected with recombinant ILF2 lentivirus compared with the control group. Consistent with these results, ILF2 downregulation increased the apoptotic cell count in the ILF2 siRNA group (Figure 3). Together, these results suggest that ILF2 might be involved in liver cancer cell proliferation and apoptosis.

### 2.4. Overexpression of Interleukin Enhancer Binding Factor 2 Promotes Tumor Growth in a Xenograft Model

**Figure 4:**
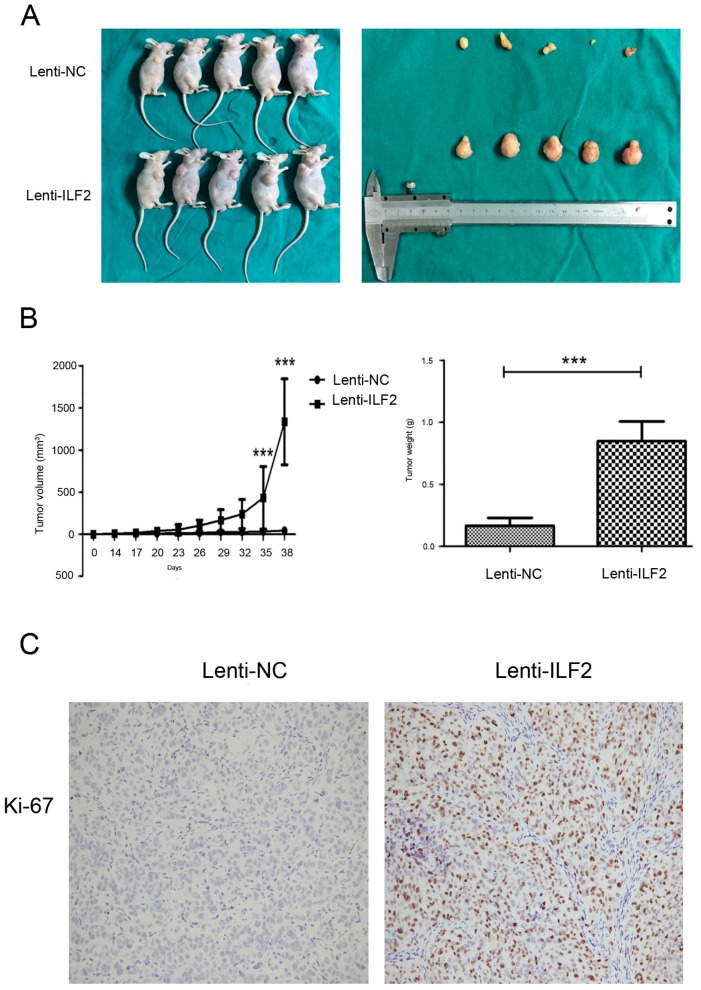
ILF2 overexpression promotes MHCC-LM3 cell tumorigenicity in vivo. (**A**) Tumors dissected from nude mice bearing Lenti-NC or Lenti-ILF2 cells; (**B**) Tumor growth curves measured after subcutaneous injection of Lenti-NC or Lenti-ILF2 cells. The tumor volume was calculated with calipers every three days for 38 days. Data shown as mean (*n* = 5) ± SD, *** *p* < 0.001; with tumor weights measured just after mice were sacrificed in the two groups; (**C**) Immunohistochemical images of Ki-67 expression. Representative images at 200× magnification.

The findings suggesting that ILF2 might play a role in apoptosis and promotion of HCC cell proliferation in vitro prompted us to confirm whether ILF2 exerts a similar effect in vivo. Lenti-NC or Lenti-ILF2 cells were subsequently implanted into the right axilla of nude mice. The mice were sacrificed five weeks after inoculation and tumors were excised, measured, and photographed (Figure 4A). Compared with the Lenti-NC group, the volume and weight of the tumors were significantly larger in mice bearing Lenti-ILF2 (Figure 4B). Consistently, the expression of Ki-67, a cell proliferation marker, was increased in nude mice after subcutaneous injection of Lenti-ILF2 cells (Figure 4C). Five weeks after inoculation, Western blot analysis revealed high expression levels of ILF2 in tumors formed from the Lenti-ILF2 group. This indicated that Lenti-ILF2 effectively overexpresses ILF2 in vivo for more than a month (Figure S3). Taken together, these results demonstrate the ability of ILF2 to modulate the proliferative capacity of liver cancer cells in vivo.

### 2.5. Interleukin Enhancer Binding Factor 2 May Suppress Apoptosis in Tumors via Regulation of Pro-Apoptotic Proteins and Anti-Apoptotic Proteins

**Figure 5:**
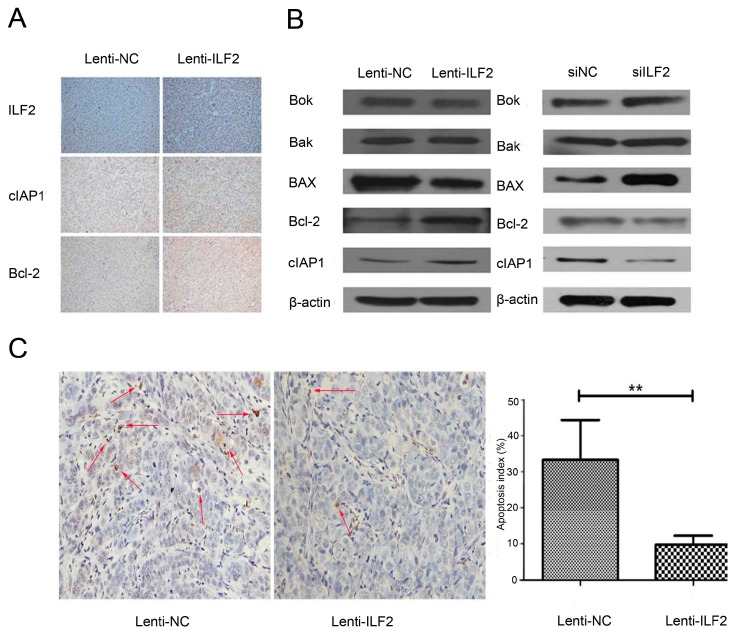
ILF2 regulates the expression of apoptosis-related proteins in xenografted tumors and liver cancer cells. (**A**) Immunohistochemistry of ILF2, cellular inhibitor of apoptosis 1 (cIAP1) and B-cell lymphoma 2 (Bcl-2) in xenografted Lenti-NC and Lenti-ILF2 tumors. Representative images at 100× magnification; (**B**) Effect of ILF2 overexpression and downregulation on apoptosis-related protein expression in cancer cells infected with Lenti-ILF2 or transfected with siILF2, respectively. Relative band intensity from Western blot analysis was normalized by the expression level of β-actin; (**C**) Terminal deoxynucleotidyl transferase dUTP nick end labeling (TUNEL) staining of xenografted tumors in the Lenti-NC and Lenti-ILF2 groups. Representative images at 200× magnification. Red arrows indicate apoptotic cells. Data shown as mean (*n* = 3) ± SD, ** *p* < 0.01.

We next explored the molecular mechanism underlying ILF2 apoptosis suppression. As B-cell lymphoma 2 (Bcl-2) is a key regulator of apoptosis and ILF2 enhances IRES-dependent translation of endogenous cIAP1 [11], the expression of anti-apoptotic Bcl-2 and cIAP1 were measured in nude mice bearing Lenti-ILF2 or Lenti-NC cells. Interestingly, immunohistochemistry proved that Bcl-2 and cIAP1 levels were significantly increased in tumors from Lenti-ILF2-treated mice compared with the control group (Figure 5A). To further confirm the relationship between ILF2 and the expression of pro-apoptotic and anti-apoptotic proteins, we analyzed the levels of pro-apoptotic proteins such as Bcl-2-associated X protein (Bax), Bcl-2 antagonist/killer 1 (Bak), and Bcl-2 related ovarian killer (Bok); and anti-apoptotic proteins such as Bcl-2 and cIAP1 by Western blot in cancer cells infected with Lenti-ILF2 and Lenti-NC or transfected with ILF2 siRNA (siILF2) and siRNA negative control (siNC). Bcl-2 and cIAP1 protein levels were significantly upregulated, and levels of Bax and Bok were downregulated in Lenti-ILF2 cells; similar results were also validated in siILF2 cells, but there were no changes on Bak levels in cancer cells infected with Lenti-NC and Lenti-ILF2 or transfected with siNC and siILF2 (Figure 5B). The results were further confirmed by terminal deoxynucleotidyl transferase dUTP nick end labeling (TUNEL) assay, which was employed to detect apoptosis in xenograft tumors. As shown in Figure 5C, the control group revealed more extensive apoptosis compared with cells from Lenti-ILF2 xenografts. These results suggest that ILF2 might suppress apoptosis in liver cancer cells via regulation of pro-apoptotic proteins and anti-apoptotic proteins in vivo and in vitro.

## 3. Discussion

HCC has fairly high morbidity and is difficult to treat, with very poor prognosis if not diagnosed early. Despite decades of study on its etiology, pathogenesis, and epidemiology, targeted agents (Sunitinib) have currently failed to provide meaningful improvements in the prognosis of HCC. Increasing evidence indicates that the majority of tumors show enhanced expression of ILF2 compared with their normal healthy counterparts [18,19,20,21]. Thus, we focused on the correlation between ILF2 expression and outcome in HCC patients, and its role in HCC proliferation.

ILF2 is a nucleic acid-binding protein that binds to ILF3 [6]. Besides binding to double-stranded (ds) RNA, the ILF2–ILF3 complex has the ability to bind to single-stranded and dsDNA [26]. In addition, ILF2 may be an important interacting factor for DNA-dependent protein kinase (DNA-PK), which plays a key role in DNA double-strand break repair [27]. It has been reported that ILF2 might be highly expressed in HCC specimens, compared to the normal liver, and serves as a potential molecular target of HCC [23], in agreement with their results, we also showed that higher ILF2 expression was observed in most HCC cell lines as well as HCC tissues from patients (Figure 1). To explore whether ILF2 upregulation occurs via transcription and/or degradation, the Huh7 and MHCC-LM3 cells were incubated with the proteasome inhibitor MG132 for various times, and our results revealed that ILF2 upregulation happens via transcription and not via degradation of ILF2 or protein turnover (Figure 1F). In addition to HCC, increased ILF2 expression has also been detected in glioma, non-small cell lung cancer, and esophageal cancer [18,19,20]. Therefore, overexpression of ILF2 might provide a beneficial effect to tumor cells during tumor progression. Moreover, a recent report has indicated a close correlation between ILF2 expression and tumor size in pancreatic ductal adenocarcinoma (PDAC) and that ILF2 could be a valuable prognostic indicator for survival in PDAC patients [28]. Consistent with these findings, we found a significant association between ILF2 expression and tumor size (Table 1). HCC patients with high ILF2 expression have dismal clinical outcomes from TCGA data analysis, strongly supporting the clinical relevance of ILF2 in HCC prognosis.

Loss of proliferation control is a hallmark of cancer, including liver cancer. Cells fail to proliferate in part due to apoptosis. Apoptosis, the first identified programmed cell death process, has been widely investigated in the chemical treatment of tumors [29]. Evasion of apoptosis, a characteristic of tumor cells, occurs by altering the expression levels and functions of apoptosis regulators [30]. In apoptosis, the mitochondrial outer membrane permeabilization (MOMP) is considered as a “point of no return” and is tightly regulated by the well-known Bcl-2 family proteins [31]. Other apoptosis regulators frequently changed in tumors are the inhibitors of apoptosis proteins (IAPs). Our results showed that ILF2 could regulate cell proliferation and apoptosis in vitro (Figure 2 and Figure 3), which is consistent with our hypothesis. In vivo, overexpression of ILF2 could promote tumor growth in a xenograft model (Figure 4). The findings prompted us to search for ILF2 target genes involved in cell proliferation and apoptosis regulation. ILF2 has been shown to regulate IRES activity and translation of the human AU-rich IRES-containing mRNAs of cIAP1, XIAP, the E26 transformation-specific (ETS) family transcription factor ELG, and nuclear respiratory factor (NRF) [7]. Also, ILF2 enhances translation of endogenous cIAP1 mRNA, and ILF2-dependent translation of cIAP1 is mediated by its IRES [8]. The expression and functions of IAPs have been reported in various human cancers, such as esophageal, colon, cervical, and prostate cancer [32,33,34,35]. It has been reported that the biological activities exerted by ILF2 are mediated by apoptosis pathways [22,36]. Furthermore, Bcl-2 and cIAP1 are involved in apoptosis and proliferation processes in HCC [37,38]. Here we show that ILF2 might regulate Bcl-2 and cIAP1 expression in xenografted tumors and liver cancer cells, suggesting that the oncogenic function of ILF2 was at least in part by promoting the expression of Bcl-2 and cIAP1 (Figure 5B). In addition to these two anti-apoptotic proteins, it also regulates the expression of pro-apoptotic proteins, such as Bax and Bok. Notably, the control group showed more extensive apoptosis compared with cells from Lenti-ILF2 xenografts by TUNEL assay (Figure 5C). The findings indicated that Bcl-2, Bok, Bax, and cIAP1 are likely to mediate the effect of ILF2 on apoptosis regulation in vivo and in vitro. However, Bak expression levels are not affected in the overexpression or knockdown groups, probably because Bak is not critically involved in the process of ILF2-induced cell proliferation or apoptosis. Furthermore, we proved that the expression of Ki-67, a cell proliferation marker, was upregulated in nude mice bearing Lenti-ILF2 cells (Figure 4C). Huang et al. [18] also reported that the expression of NF45 positively and significantly correlates with Ki-67 expression in glioma patients. It has been previously reported that repression of either ILF2 or its binding partner ILF3 leads to retardation of HeLa cell growth and accumulation of multinucleate giant cells [39]. In addition, ILF3 is upregulated in HCC and its expression increases HCC growth both in vitro and in vivo [40]. Similar to that observation, our results showed upregulation of ILF2 in HCC and that ILF2 plays a significant role in liver cancer cell growth in vitro and in vivo. Therefore, elucidating the molecular mechanisms underlying ILF2 ability to induce cancer cell proliferation and whether this is mediated by ILF2–ILF3 interactions in HCC pathogenesis, requires further study.

In conclusion, our work shows that ILF2 expression is associated with cell proliferation and apoptosis progression in vitro and in vivo. Moreover, our findings shed light into the mechanisms by which ILF2 influences apoptosis progression via regulation of Bcl-2, Bok, Bax, and cIAP1; with cIAP1 acting as a key regulatory protein that communicates between apoptotic and cell proliferation pathways. Birinapant is a specific cIAP1 inhibitor that could potentially be used in HCC treatment. Therefore, the features of this ILF2–cIAP1 crosstalk support its exploration as a potential therapeutic target for HCC. The ILF2–ILF3 complex, is involved in several steps of RNA metabolism [41]. Thus, the downstream signaling pathways and interactions between ILF2 andILF3 in tumorigenesis require further investigation.

## 4. Materials and Methods

### 4.1. Cell Lines and Cultures

All cell lines were purchased from the Shanghai Institute of Cell Biology, Chinese Academy of Sciences (Shanghai, China). Cells were cultured in Dulbecco’s modified Eagle medium (Gibco, Grand Island, NY, USA) supplemented with 10% fetal bovine serum (FBS), 100 U/mL of penicillin and streptomycin, and maintained at 37 °C in a humidified 5% CO_2_ incubator.

### 4.2. Lentiviral Transfection and siRNA Knockdown

A lentiviral vector carrying Flag-ILF2 or a negative control vector and corresponding viruses (1 × 10^8^ pfu/mL) were constructed and prepared by GeneChem Co., Ltd. (Shanghai, China). Lentiviral transfection was carried out in the presence of polybrene (GeneChem Co., Ltd.) according to the manufacturer’s guidelines. The siIFL2 for ILF2 knockdown and siNC as a negative control were purchased from Invitrogen (Cat. No. 1299001; Waltham, MA, USA). Transfection was performed using lipofectamine 2000 transfection reagent (Invitrogen) and Opti-MEM (Thermo Fisher, Waltham, MA, USA), as the datasheet suggests.

### 4.3. Terminal Deoxynucleotidyl Transferase dUTP Nick-End Labeling Assay

Apoptosis in xenograft tumors was also measured using the TUNEL assay, which was conducted using the Trevigen Apoptotic Cell Sysem (TACS) XL-Blue label in situ apoptosis detection kit (Trevigen, Gaithersburg, MD, USA) according to the manufacturer’s guidelines. Briefly, the sections were rinsed in water and then equilibrated with phosphate-buffered saline (PBS). The specimens were permeabilized with 0.1% Triton X-100 for 2 min on ice. After rinsing, a reaction buffer was used to incubate the sections in a humidified chamber. Next, visualization of the reaction was carried out using TACS Blue Label solution.

### 4.4. Immunohistochemistry

The formalin-fixed paraffin-embedded tissues were deparaffinized and rehydrated. Antigen repair was conducted in 10 mM citric acid buffer (pH 6.0) for 10 min using a microwave oven. Endogenous peroxidase activity was blocked by 3% hydrogen peroxide in methanol for 20 min at room temperature. The sections were further incubated with Dako Liquid DAB Large-Volume Substrate-Chromogen System (Dako, Glostrup, Denmark). After counterstaining with Mayer’s hematoxylin, the slides were dehydrated, cleared, and mounted. Immunostaining was evaluated by an Olympus BX-50 light microscope (Olympus, Tokyo, Japan). The stain density was analyzed using an Image Pro-Plus 6.0 analysis system (Media Cybernetics Inc., Silver Spring, MD, USA).

### 4.5. Cell Viability Assay

A cell counting kit (CCK-8, Dojindo Molecular Technologies, Inc., Kumamoto, Japan) was used to detect cell viability. Briefly, 3000 viable cells per well were seeded into 96-well culture plates. Each plate was subjected to the CCK-8 assay according to the manufacturer’s protocol. After a 2 h incubation at 37 °C, the relative viable cell numbers were measured by the absorbance optical density at 450 nm using a microplate reader (BioTek, Winooski, VT, USA). The results represented the mean ± standard deviation (SD) of three independent experiments.

### 4.6. Western Blotting

Protein concentration was measured using a BCA Protein Assay Kit (Pierce, Rockford, IL, USA). Proteins were denatured by heating at 90 °C for 10 min in 4× nuPAGE LDs sample buffer (Life Technologies, Carlsbad, CA, USA). Equal amounts of protein from each sample were separated by 6%–18% sodium dodecyl sulfate-polyacrylamide gel electrophoresis (SDS–PAGE) (Life Technologies). Then, the proteins were transferred to a polyvinylidene fluoride membrane (Millipore, Billerica, MA, USA). After blocking with 5% non-fat milk, the membrane was incubated overnight at 4 °C with the primary anti-ILF2 antibody (1:1000; HPA007484; Sigma-Aldrich, St. Louis, MO, USA), anti-Flag (1:1000; SAB4200071; Sigma-Aldrich), anti-Bcl-2 (1:1000; #15071; Cell Signal, Danvers, MA, USA), anti-cIAP1 (1:1000; ab108361; Abcam, Cambridge, MA, USA), anti-Bax (1:1000; #5023; Cell Signal), and anti-β-actin (1:1000; #3700; Cell Signal) overnight. After washing three times with Tris-buffered saline with 0.05% Tween-20 for 10 min each, signal was detected with the SuperSignal West Pico Chemiluminescent Substrate (Pierce). The band signals were quantified using the Image J 1.44 software from Wayne Rasband (National Institutes of Health, Bethesda, MD, USA). β-Actin was used as a protein control.

### 4.7. Colony Formation Assay

Liver cancer cells were seeded in six-well plates at a density of 500 cells per well and continually cultured at 37 °C in a humidified 5% CO_2_ incubator for two weeks.

After incubation, the supernatants were discarded, and cells were rinsed three times with PBS. The cells were fixed with methanol for 15 min and stained with 0.1% crystal violet for 10 min. Colony numbers containing more than 50 cells were manually counted. The experiments were performed in triplicate.

### 4.8. Tumor Xenografts

MHCC-LM3 cells were plated and infected with lentivirus carrying either Lenti-ILF2 or Lenti-NC at multiplicity of infection (MOI) of 100. Cells were subcutaneously injected into the right anterior armpit of six-week-old female BALB/c nude mice. Tumor volume was calculated using a caliper every three days. Mice were sacrificed after tumor inoculation for five weeks, and the volume and weight of each tumor were measured. Xenograft tumors were fixed in 10% buffered formalin and embedded in paraffin for immunohistochemical staining assay. All experimental procedures were performed in accordance with the Guide for the Care and Use of Laboratory Animals and approved by our institutional ethical guidelines (The project code: 2016-294, Date: 2016-08-16) for animal experiments.

### 4.9. Apoptosis Assay

Apoptosis was detected by flow cytometry, measured by annexin V-fluorescein isothiocyanate (FITC) and propidium iodide (PI) (BD Bioscience, San Jose, CA, USA). Experimental procedures were performed as described by the manufacturer’s guidelines. After incubation with 5 μL of annexin V and 5 μL of PI at room temperature in the dark for 15 min, stained cells were analyzed in a flow cytometer model BD FACS Canto II (BD Biosciences, San Diego, CA, USA), and 10,000 events were analyzed by sample.

### 4.10. Statistical Analysis

Measurement data were presented as mean ± SD from at least three independent experiments. The comparisons between the two groups were analyzed by Student’s *t*-test. The statistical analysis was performed using GraphPad Prism software 5.0 (GraphPad Software Inc., La Jolla, CA, USA) and SPSS 15.0 (IBM, Chicago, IL, USA). All tests were two-tailed, and a *p* value of less than 0.05 was considered statistically significant.

## Figures and Tables

**Table 1:** Correlation of ILF2 expression with the observed clinicopathological features of 72 HCC patients.

Variable	Patients (Total = 72)		ILF2		*p*-Value ^a^
			Negative	Positive	
Gender	Male	61	24	37	
	Female	11	6	5	0.347
Age (years)	≥50	42	16	26	
	<50	30	14	16	0.467
Tumor size (cm)	≥10	24	6	18	
	<10	48	24	24	0.043 *
TNM stage	I–II	41	15	26	
	III–IV	31	15	16	0.315
Vascular invasion	Yes	23	11	12	
	No	49	19	30	0.468
Histopathologic grading	Good/moderate	45	18	27	
	Poor	27	12	15	0.711
Cirrhosis	Present	27	11	16	
	Absent	45	19	26	0.902

^a^ Statistical analyses were performed with chi-square test or Fisher’s exact test; * *p* < 0.05.

## Supplementary Materials

Supplementary materials can be found at www.mdpi.com/1422-0067/17/8/1373/s1.
