# Supplementary material for: Expression and Critical Role of Interleukin Enhancer Binding Factor 2 in Hepatocellular Carcinoma
Source: Int J Mol Sci. 2016 Aug 22;17(8):1373. doi: 10.3390/ijms17081373 (PMC5000768; doi:10.3390/ijms17081373)
Supplement: Supplementary file 1 [file ijms-17-01373-s001.pdf]

# Supplementary Materials: Expression and Critical Role of Interleukin Enhancer Binding Factor 2 in Hepatocellular Carcinoma

**Shaobing Cheng, Xu Jiang, Chaofeng Ding, Chengli Du, Kwabena Gyabaah Owusu-Ansah, Xiaoyu Weng, Wendi Hu, Chuanhui Peng, Zhen Lv, Rongliang Tong, Heng Xiao, Haiyang Xie, Lin Zhou, Jian Wu and Shusen Zheng**

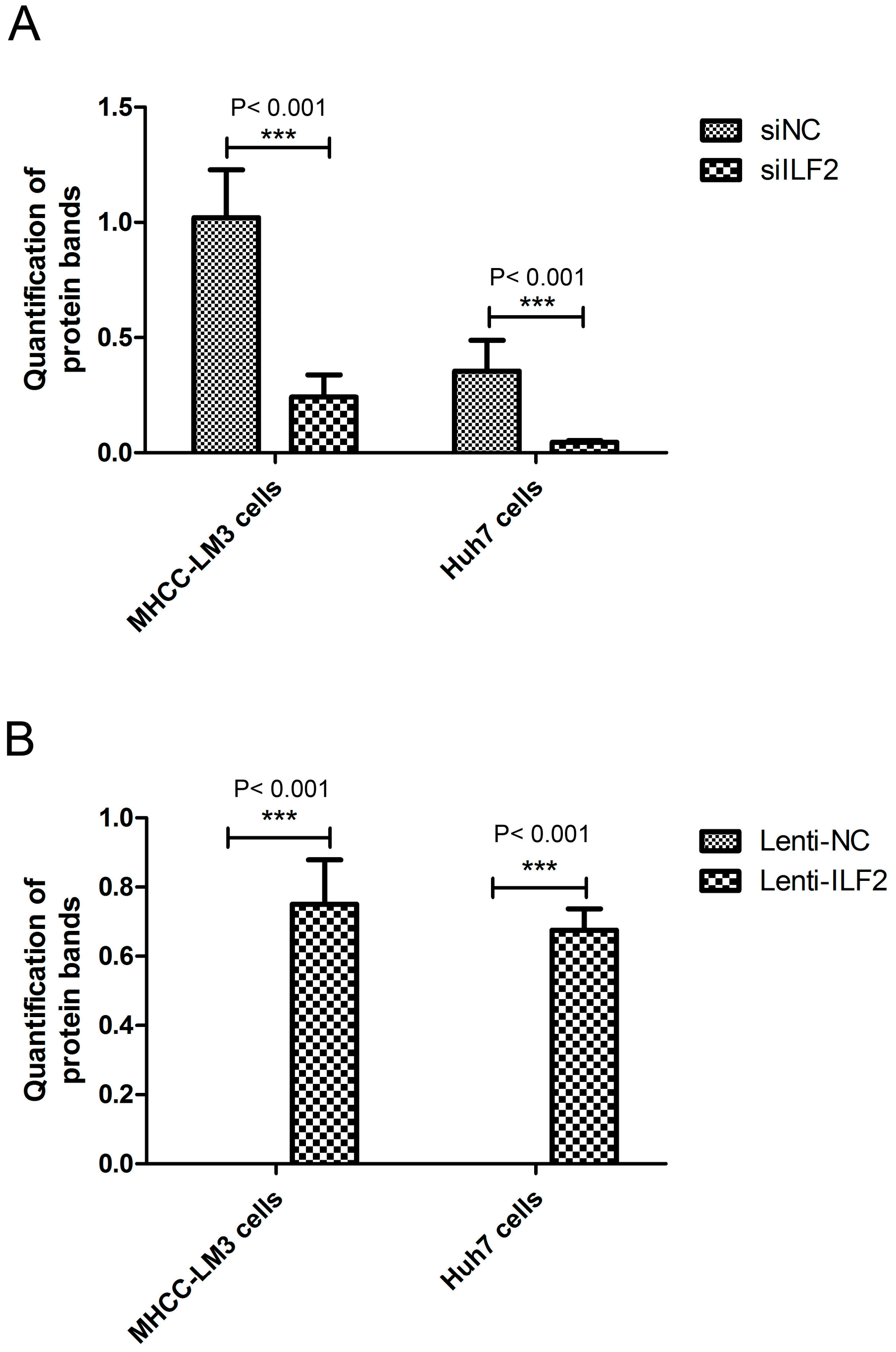


**Figure S1.** Detected Western blot ILF2 bands compared to β-actin. (**A**) Densitometric quantification of Western blot ILF2 protein bands in cancer cells transfected with siNC and siILF2 or (**B**) infected with Lenti-NC and Lenti-ILF2.

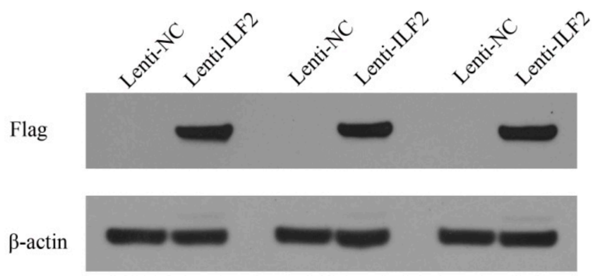


**Figure S2.** Expression of exogenous ILF2 in liver cancer cells validated and Flag-ILF2 detected by anti-Flag antibody in Lenti-ILF2 cells.

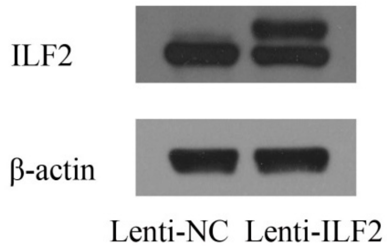


**Figure S3.** Western blot analysis showing high ILF2 expression in the Lenti-ILF2 group.
